# Catalytic inhibitors of DNA topoisomerase II suppress the androgen receptor signaling and prostate cancer progression

**DOI:** 10.18632/oncotarget.4105

**Published:** 2015-05-12

**Authors:** Haolong Li, Ning Xie, Martin E. Gleave, Xuesen Dong

**Affiliations:** ^1^ The Vancouver Prostate Centre, Department of Urologic Sciences, University of British Columbia, Canada

**Keywords:** topoisomerase II, catalytic inhibitor, androgen receptor, prostate cancer, castration resistant prostate cancer

## Abstract

Although the new generation of androgen receptor (AR) antagonists like enzalutamide (ENZ) prolong survival of metastatic castration-resistant prostate cancer (CRPC), AR-driven tumors eventually recur indicating that additional therapies are required to fully block AR function. Since DNA topoisomerase II (Topo II) was demonstrated to be essential for AR to initiate gene transcription, this study tested whether catalytic inhibitors of Topo II can block AR signaling and suppress ENZ-resistant CRPC growth. Using multiple prostate cancer cell lines, we showed that catalytic Topo II inhibitors, ICRF187 and ICRF193 inhibited transcription activities of the wild-type AR, mutant ARs (F876L and W741C) and the AR-V7 splice variant. ICRF187 and ICRF193 decreased AR recruitment to target promoters and reduced AR nuclear localization. Both ICRF187 and ICRF193 also inhibited cell proliferation and delayed cell cycling at the G2/M phase. ICRF187 inhibited tumor growth of castration-resistant LNCaP and 22RV1 xenografts as well as ENZ-resistant MR49F xenografts. We conclude that catalytic Topo II inhibitors can block AR signaling and inhibit tumor growth of CRPC xenografts, identifying a potential co-targeting approach using these inhibitors in combination with AR pathway inhibitors in CRPC.

## INTRODUCTION

Androgen deprivation therapy (ADT) is the first line of treatment for locally advanced, recurrent or metastatic prostate cancers. It aims at suppressing transcriptional activity of the androgen receptor (AR). Despite frequent and often durable responses, progression to castration-resistant prostate cancer (CRPC) invariably occurs, most often driven by reactivation on the AR pathway via mechanisms involving AR amplification, overexpression, mutations as well as intratumoral steroidogenesis [1–3]. These observations indicate that cellular resistance to ADT is not necessarily a result of the acquisition of growth independence from androgens, as was previously believed, but rather reflect cellular acquisition of mechanisms to overcome castrate-levels of androgens. Recently, more potent androgen receptor (AR) pathway inhibitors like abiraterone and enzalutamide (ENZ) improved survival in metastatic CRPC [4, 5]. However, resistance emerges even with the most potent AR pathway inhibitors [4, 5]. These observations emphasize that alternative approaches are required to thoroughly suppress AR signaling in patients with CRPC. Genome-wide profiling studies demonstrated that the AR regulated transcriptome in CRPC is significantly different from that in ADT-naïve prostate cancers [6]. AR activated genes in CRPC were dominated by cell cycle and mitosis genes such as UBE2C, CDC20 and CDK1 [6, 7]. These results suggest that targeting cell mitosis controlled by the AR signaling in CRPC may inhibit tumor growth and progression more effectively.

The transcriptional activity of AR requires DNA Topoisomerase II (Topo II) to be recruited to target promoters [8–10]. Topo II creates transient, but fixable DNA double strand breaks to relax the topology of DNA, which are required for AR to mediate transcription initiation [9, 10]. Blocking Topo II expression or function impairs AR transcriptional activity and AR-driven cell proliferation [8]. Additionally, Topo II was also demonstrated to be responsible for gene fusion of TMPRSS2-ERG, one of the most common genomic alterations in prostate cancer [8, 10]. Increased expression of Topo II is associated with higher Gleason score and relative ADT insensitivity of prostate tumors [11]. These findings suggest that inhibition of both Topo II and AR may cooperatively de-activate AR signaling and delay CRPC progression.

There are two types of Topo II inhibitors, Topo II poisons and catalytic inhibitors. Topo II poisons are exemplified by the cytotoxic chemotherapeutic, etoposide. When DNA breaks are created by Topo II, etoposide inhibits Topo II to re-ligate DNA breaks resulting in DNA damage. Such unfixable DNA damages cause cell cycle arrest and subsequently apoptosis [12–14]. Catalytic Topo II inhibitors are exemplified by ICRF187 and ICRF193, which target the catalytic domain of Topo II and prevent formation of DNA double strand breaks, resulting in unrelaxed DNA conformation [15]. Additionally, ICRF193 was reported to induce cell cycle arrest by inhibiting chromosome condensation and segregation at the M phase of cell cycle, as Topo II is required to de-catenate the intertwined chromosomes [16]. Unlike Topo II poisons, catalytic inhibitors block Topo II activity without creating DNA breaks, and hence exhibit minimal cytotoxic effects. Together, these findings led us to hypothesize that catalytic Topo II inhibitors may block AR signaling and induce G2/M cell cycle arrest in prostate cancer cells.

In this study, we demonstrate that catalytic Topo II inhibitors can block AR signaling and inhibit prostate cancer cell proliferation and CRPC growth, provide proof-of-principle that co-targeting Topo II in combination with AR pathway inhibitors may cooperatively de-activate the AR and delay CRPC progression.

## RESULTS

### Topo II is required for the transcriptional activity of the AR

**Figure 1:**
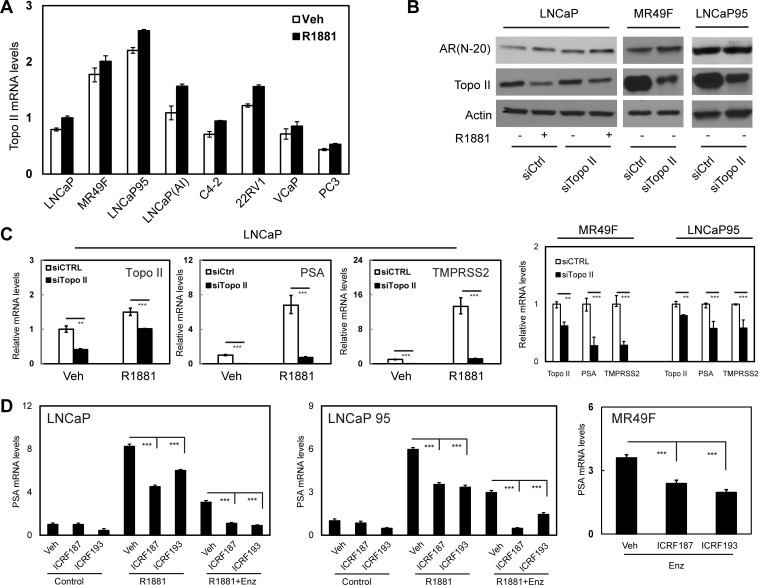
Blocking Topo II expression or inhibiting Topo II activity represses AR transcriptional activity (**A**) LNCaP, MR49F, LNCaP95, LNCaP(AI), C4-2, 22RV1, VCaP and PC3 cells were cultured in mediums containing 5% CSS for 48h and then treated with vehicle or 1nM of R1881 for 24 hours. (**B**–**C**) Relative Topo II beta mRNA levels to GAPDH were measured by real-time PCR. LNCaP, MR49F and LNCaP95 cells were transfected with control or Topo II beta siRNA, and then treated with vehicle or 1nM of R1881 for 24 hours. AR and Topo II beta protein were detected by immunoblotting (B). Relative mRNA levels of Topo II beta, PSA and TMPRSS2 to GAPDH were measured by real-time PCR (C). (D) LNCaP, LNCaP95 and MR49F cells were cultured in RPMI1640 medium containing 5% CSS. Cells were treated with vehicle, 1nM of R1881 or 1nM of R1881 plus 5uM of ENZ for 24 hours. Cells were also co-treated with DMSO, 1uM of ICRF187 or 1uM of ICRF193 as indicated. Relative RNA levels of PSA to GAPDH were measured by real-time PCR from three independent experiments. Data represent mean ± SEM (*n* = 3) with *P* < 0.01 as ** and *P* < 0.001 as *** (student's *t*-test).

Two Topo II isoforms, alpha and beta, are widely expressed in prostate cancer cells [17]. We confirmed that Topo II beta was highly expressed in our collection of human prostate cancer cell lines (Figure 1a). Androgen-independent LNCaP(AI) and LNCaP95 cell lines as well as ENZ-resistant MR49F cell line expressed higher levels of Topo II beta than their parental LNCaP cells. Androgen treatment did not significantly alter Topo II beta expression. In LNCaP cells, Topo II beta silencing dramatically reduced mRNA levels of AR regulated genes including PSA and TMPRSS2 (Figure 1b–1c). These results were also confirmed in MR49F and LNCaP95 cells (Figure 1c). Reduced mRNA expression of PSA and TMPRSS2 by Topo II silencing was not due to reduced AR protein levels in these cells (Figure 1b).

ICRF187 and ICRF193 are catalytic inhibitors to both Topo II isoforms [18]. We treated prostate cancer cells with these inhibitors to determine their impacts on AR signaling by measuring AR targeted transcription of PSA, TMPRSS2 and FKBP5 genes (Figure 1d and S2a). Although LNCaP and LNCaP95 cells responded to 1nM of R1881 by upregulating PSA, TMPRSS2 and FKBP5 expression, both ICRF187 and ICRF193 caused 30-60% deduction in mRNA levels of these genes. Similar results were also obtained in VCaP cells (Figure S2b). Moreover, ICRF187 and ICRF193 further inhibited PSA mRNA levels when LNCaP cell were under 5uM of ENZ (Figure 1d and S2). Importantly, ICRF187 and ICRF193 suppressed PSA mRNA levels by 40-50% in ENZ-resistant MR49F cells (Figure 1d).

### ICRF187 and ICRF193 block transcriptional activity of AR, AR mutants, and AR-V7 in prostate cancer cells

**Figure 2:**
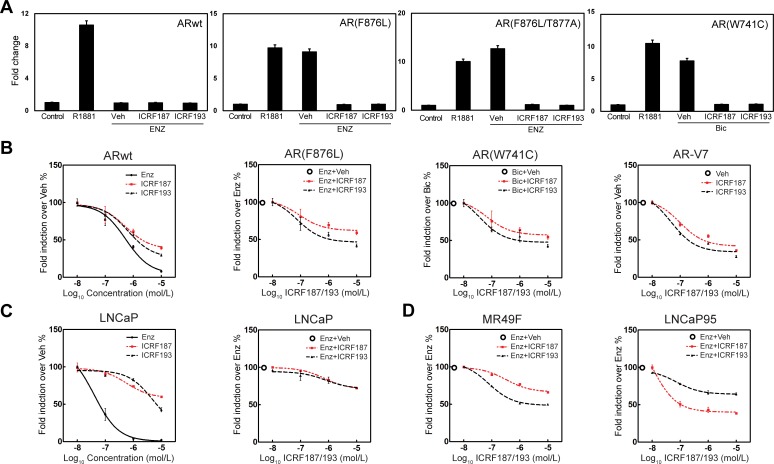
Topo II catalytic inhibitors suppress AR mutant and AR-V7 transcriptional activities (**A**) 293T cells were transfected with a PSA-luciferase reporter plus expression plasmids encoding the wild type AR, AR(F876L), AR(W741C) or AR-V7. Cells were treated with DMSO, 1nM of R1881, 10uM of ENZ or bicalutamide, 10uM of ENZ or Bicalutamdie plus 1uM of ICRF187 or ICRF193 for 24 hours. (**B**) 293T cells were cultured in medium containing 10% FBS, transfected with wild type AR and treated with vehicle, 0.01-10uM of ICRF187, ICRF193 or ENZ. 293T cells transfected with AR(F876L) were treated with 10uM of ENZ plus vehicle, 0.01-10uM of ICRF187 or ICRF193. 293T cells transfected with AR(W741C) were treated with 10uM of bicalutamide plus vehicle, 0.01-10uM of ICRF187 or ICRF193. PC3 cells transfected with AR-V7 were treated with vehicle, 0.01-10uM of ICRF187 or ICRF193. (**C**) LNCaP cells were transfected with a PSA-luciferase reporter and treated with 1nM of R1881. Cells were also treated with 0.01-10uM of ENZ, ICRF187 or ICRF193 (*left panel*). Cells were treated with 5uM of ENZ plus 0.01-10uM of ICRF187 or ICRF193 (*right panel*). (**D**) MR49F and LNCaP95 cells were transfected with a PSA-luciferase reporter. MR49F cells were treated with 10uM of ENZ plus 0.01-10uM of ICRF187 or ICRF193, while LNCaP95 cells were treated with 5uM of ENZ plus 0.01-10uM of ICRF187 or ICRF193 for 24 hours. Relative luciferase activities were calibrated with Renilla from three independent experiments and were presented as mean ± SEM (*n* = 3). Values from vehicle treatment were set as 100%.

We next tested the effect of catalytic Topo II inhibitors on the transcriptional activity of mutant AR and the AR-V7 splice variant. The AR carrying the F876L mutation can be transcriptionally activated by ENZ, while the W741C mutation can activate the AR by bicalutamide [19, 20]. In AR negative 293T cells, PSA-luciferase reporter activities driven by AR(F876L) or AR(F876L/T877A) in the presence of 10uM of ENZ were significantly suppressed by ICRF187 or ICRF193 (Figure 2a). Similarly, ICRF187 and ICRF193 also inhibited the luciferase activity driven by AR(W741C) in the presence of bicalutamide. Additionally, luciferase activity driven by AR-V7 was also strongly repressed by ICRF187 and ICRF193 dose-dependently in AR negative PC3 cells (Figure 2b). When LNCaP cells were transfected with the PSA-luciferase reporter and treated with 1nM of R1881 plus 0.01-10uM of ENZ, ICRF187 or ICRF193, we observed that ENZ potently suppressed the transcriptional activity of AR-FL. ICRF87 and ICRF193 also showed dose-dependent inhibition of luciferase activity but to milder extents (Figure 2c). However, when LNCaP cells were co-treated with 5uM of ENZ plus 0.01-10uM of either ICRF187 or ICRF193, a further 20% deduction in luciferase activity was observed. These results suggest that Topo II inhibitors can increase the efficacy of ENZ in blocking the AR function. In ENZ-resistant MR49F, ICRF187 and ICRF193 resulted in 40% and 60% decrease in luciferase activity respectively (Figure 2d). LNCaP95 cells express not only full length AR, but also AR splice variants including AR-V7 and AR_v567ed_. Co-treatment of ICRF187 or ICRF193 with 5uM of ENZ induced a further 60% decrease in luciferase activity driven by the AR splice variants in LNCaP95 cells (Figure 2d). Interestingly, several other catalytic inhibitors of Topo II including Merbarone, Aclacinomycin A and Genistein all showed suppressive impacts to AR signaling in prostate cancer cells (Figure S3). By contrast, the Topo II poison, etoposide, increased AR transactivation dose-dependently. Together, these results demonstrate that catalytic Topo II inhibitors can repress AR transcriptional activity in prostate cancer cells.

### ICRF187 and ICRF193 impair DNA binding and nuclear localization of the AR

**Figure 3:**
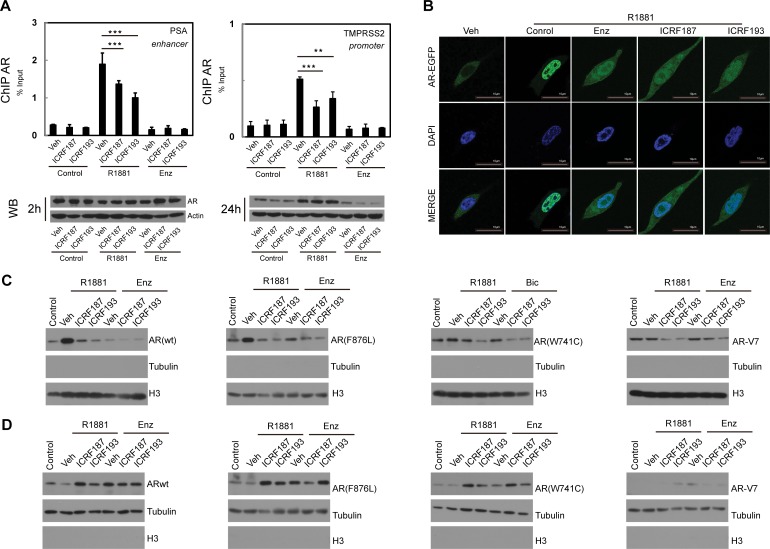
ICRF187 and ICRF193 inhibit AR recruitment to target promoters and AR nuclear localization (**A**) LNCaP cells were cultured in RPMI1640 medium containing 5% CSS and treated with vehicle, 1uM of ICRF187 or 1uM of ICRF193 in addition to vehicle, 10nM of R1881 or 10uM of ENZ treatment for 2 hours. Three independent ChIP experiments were performed using the AR antibody. Precipitated DNA fragment were used as templates to amplify the PSA enhancer and the TMPRSS2 promoter by real-time PCR. Data represented mean ± SEM (*n* = 3) and plotted as percentage of input. *P* < 0.01 ** and *P* < 0.001 as *** (student's *t*-test). AR protein levels under 2 and 24 hour treatment were detected by Western blotting. (**B**) LNCaP cells expressing EGFP-AR were cultured in RPMI1640 medium containing 5% CSS. Cells were treated with vehicle, 10nM of R1881, 10nM of R1881 plus 10 μM of ENZ, 10nM of R1881 plus 1uM of ICRF187, or 10nM of R1881 plus 1uM of ICRF193 for 6 hours. Cells were then fixed with 4% paraformaldehyde and mounted with DAPI. Representative confocal microscopic images showed AR localization (*Green*) and nucleus (*Blue*). (**C**–**D**) 293T cells were transfected with plasmids encoding wild type AR, AR(F876L), AR(W741C) and AR-V7. Cells were treated with vehicle, 1uM of ICRF187 or 1uM of ICRF193 in addition to 10nM of R1881, 10uM of ENZ or 10uM of bicalutamide for 24 hours. Nuclear (C) and cytosol (D) protein extract were immunoblotted with AR, tubulin and Histone H3 antibodies. Three independent experiments were performed and one set of Western blotting images are presented.

To define mechanisms by which Topo II inhibitors repress AR transactivation, we performed ChIP assays (Figure 3a). Within 2 hours of R1881 treatment, AR was robustly recruited to the androgen responsive elements in PSA and TMPRSS2 promoters. However, ICRF187 or ICRF193 resulted in 30-50% reduction of AR recruitment. These changes were not due to decreased AR protein levels within the 2 hour treatment. However, co-treatment of ICRF187 or ICRF193 with ENZ for 24 hours resulted in greater deduction in AR protein levels when compared with ENZ treatment alone. LNCaP cells expressing GFP-AR were next used to study the effects of ENZ and Topo II inhibitors on subcellular localization of AR-FL. As expected, R1881 induced, while 10uM of ENZ blocked nuclear localization of AR-FL (Figure 3b). Nuclear localization of AR-FL was reduced by 1uM of ICRF187 or ICRF193, comparable with that of ENZ. In addition, we also study subcellular localizations of AR mutants and AR-V7 under catalytic Topo II inhibitor treatment by Western blotting assays (Figure 3c–3d). 293T cells were transfected with plasmids of wild type AR, AR(F876L), AR(W741C) or AR-V7 and then treated with vehicle, ICRF187, or ICRF193 in the presence of 10nM of R1881, 10uM of ENZ or 10uM of bicalutamide. ICRF187 and ICRF193 reduced protein levels of wild type AR, AR(F876L), AR(W741C) in the nuclear extracts, but increased their protein levels in cytosol fractions. However, AR-V7 protein was primarily localized in nuclear fraction. Together, these results suggest that Topo II catalytic inhibitors supress AR recruitment to its target promoters and reduce AR protein nuclear localization.

### ICRF187 and ICRF193 suppress prostate cancer cell growth and delay cell cycling at the G2/M phase

**Figure 4:**
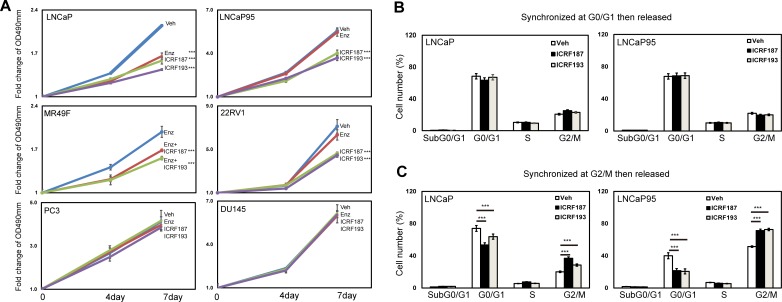
ICRF187 and ICRF193 inhibit prostate cancer cell growth and delay cell cycling at the G2/M phase (**A**) LNCaP, LNCaP95, MR49F, 22RV1, PC3 and DU145 cells were cultured in mediums containing 5% CSS for 48 hours. Cells were treated with vehicle, ENZ, 10uM of ICRF187 or 1uM of ICRF193 for 0-7 days. The dose of ENZ was 1uM in LNCaP, 5uM in LNCaP95 and 22RV1 cells and 10uM in MR49F cells. MTS assays were measured the relative cell growth rates to day 0. Results were from three independent experiments (*n* = 6/repeat). (**B**) LNCaP and LNCaP95 cells were serum starved for 12 hours and then replenished with culture medium containing serum. Treatments of vehicle, 10uM of ICRF187 or 2uM of ICRF193 were also applied to LNCaP cells for 1.5 hours or to LNCaP95 cells for 2 hours. (**C**) LNCaP and LNCaP95 cells were cultured in growth medium containing 100 ng/ml nocodazole in addition to vehicle, 10uM of ICRF187 or 2uM of ICRF193 for 12 hours. Cells were then replenished with nocodazole free medium containing vehicle, 10uM of ICRF187 or 2uM of ICRF193 for LNCaP cells for 1.5 hours or for LNCaP95 cells 2 hours. Cells were collected and used for FACS assays to determine cell populations at G0/G1, S and G2/M phases (B-C). Results were repeated from two independent experiments (*n* = 3/repeat). One-way ANOVA followed by student *t*-test was performed with *P* < 0.001 as ***.

MTS assays were used to study the effects of catalytic Topo II inhibitors on prostate cancer cell growth. Parental LNCaP cell growth was similarly inhibited by ENZ, ICRF187 or ICRF193 under androgen deprived conditions (Figure 4a). By contrast, ICRF187 and ICRF193 but not ENZ suppressed LNCaP95, MR49F cell and 22RV1 cell growth rates. Additionally, neither ENZ nor ICRF187/ICRF193 had suppressive impacts on cell proliferation of AR negative PC3 and DU145 cells. We next performed FACS assays to study the effects of ICRF187 and ICRF193 on cell cycling of prostate cancer cells. The cell cycling of LNCaP and LNCaP95 cells were first synchronized at the G0/G1 stage by serum starving and then released by adding medium containing 10% serum. We observed that neither ICRF187 nor ICRF193 altered cell population distributions during cell cycling (Figure 4b). However when cell cycle was synchronized at the G2/M phases by nocodazole and then released, ICRF187 and ICRF193 caused significant delays for the cells passing through the G2/M and entering into the G1 phase (Figure 4c). These results indicate that ICRF187 and ICRF193 inhibited cancer cell proliferation through impeding cell cycling.

### ICRF187 inhibited CRPC xenograft tumor growth

**Figure 5:**
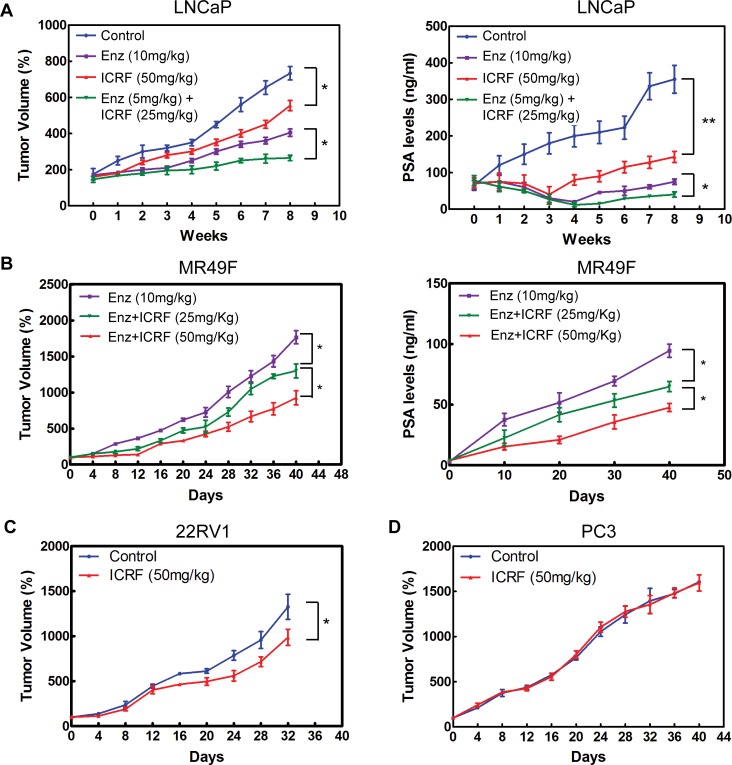
ICRF187 inhibits CRPC growth of human prostate cancer xenografts (**A**) CRPC LNCaP xenografts were treated with vehicle, 10mg/kg ENZ, 50mg/kg ICRF187 or 5mg/kg ENZ plus 25mg/kg ICRF187 (*n* = 7/group). Tumor volume and serum PSA levels were measured weekly. (**B**) Mice bearing ENZ-resistant MR49F tumors were randomly divided into three groups (*n* = 9/group) and treated with 10mg/kg ENZ, 10mg/kg ENZ plus 25mg/kg ICRF187, or 10mg/kg ENZ plus 50mg/kg ICRF187. Tumor volume and serum PSA levels were measured. (**C**) CRPC 22RV1 and (**D**) PC3 xenografts were treated with control or 50mg/kg or ICRF187. Tumor volumes were measured. Statistical analyses were performed by one-way ANOVA followed by student *t*-test with *P* < 0.05 as * and *P* < 0.01 as **.

The inhibitory effects of ICRF187 were tested in four CRPC xenograft models. After 8 weeks of treatment of CRPC LNCaP tumors, 10mg/kg daily of ENZ reduced tumor growth by 45%, compared to 24% reduction by 50mg/kg daily of ICRF187 (Figure 5a). However, combinational treatment using lower doses of ENZ (5mg/kg) and ICRF187 (25mg/kg) reduced tumor volume by 64%. Similar changes in serum PSA levels were also observed. The expression of AR targeted genes including PSA, TMPRSS2 and UBE2C as well as the tumor proliferation index Ki67 were more strongly inhibited by ENZ plus ICRF187 (Figure S4). ICRF187 inhibited ENZ-resistant MR49F xenograft growth and PSA secretion dose-dependently (Figure 5b). ICRF187 suppressed AR regulated gene expression and Ki67 index (Figure S4). Additionally, 50mg/kg of ICRF187 inhibited CRPC 22RV1 but not AR negative PC3 xenograft growth (Figure 5c–5d). These results demonstrate that ICRF187 can enhance the effects of ENZ in ENZ-sensitive LNCaP CRPC xenografts. It can also inhibit ENZ-resistant CRPC xenograft growth as monotherapy.

## DISCUSSION

Although it is well established that AR-mediated transcription initiation requires Topo II to create DNA double strand breaks at the target promoters, this fundamental knowledge has not yet been translated into effective therapies to inhibit AR signaling in CRPC patients. Our study indicates that catalytic Topo II inhibitors can block both the transcriptional activity of AR and prostate cancer cell mitosis. While re-activation of AR signaling and mitosis of cancer cells are two major features of CRPC, our study identifies catalytic Topo II inhibitors as a potential co-targeting approach in combination with AR pathway inhibitors in CRPC.

**Figure 6:**
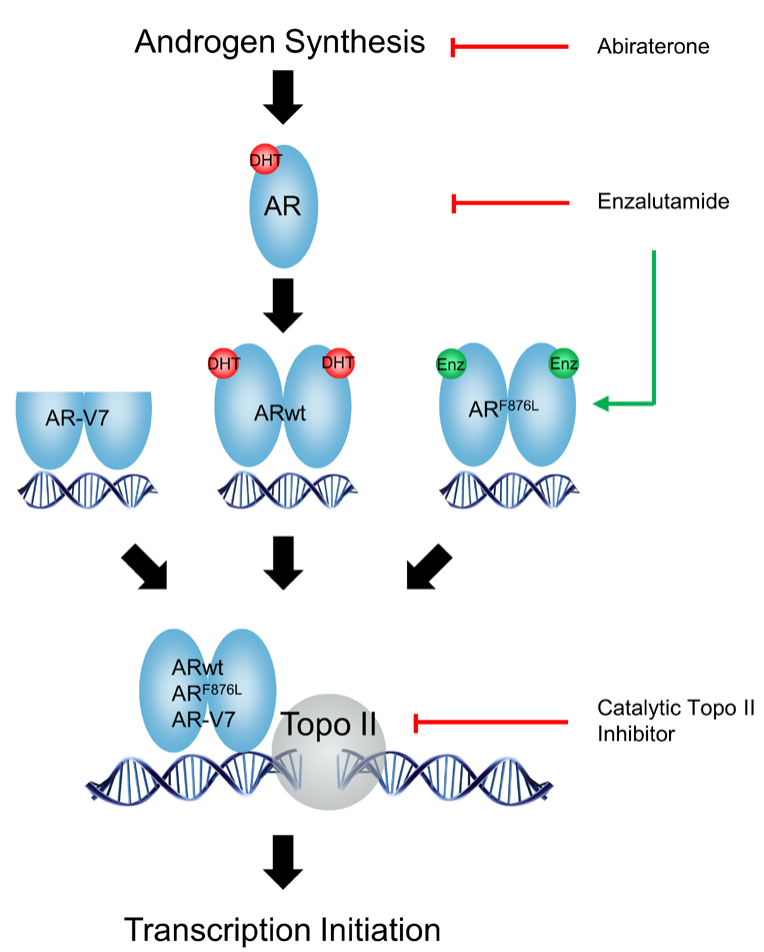
A diagram describes the mechanisms by which catalytic Topo II inhibitors and anti-AR agents block AR pathway in prostate cancer cells

ADT and newer AR pathway inhibitors such as abiraterone and ENZ aim to retain the AR in its transcriptionally inactive state, by either inhibiting androgen synthesis or antagonizing the ligand binding domain (LBD) of the AR. However, these drugs induce resistance via adaptive changes in the AR genome including AR amplification, mutations and constitutively active AR splice variants [19–21]. For example, ENZ and ARN-509 promote accumulation of the F876L point mutation in the AR which converts antagonist to agonist function and confers anti-AR resistance [19, 20]. These studies highlight that targeting the LBD of AR will have limited success due to activation of adaptive survival pathways that compromise the effectiveness of AR blockade. Our studies here present an alternative strategy, whereby targeting AR-mediated gene transcription initiation may enhance castration therapy regardless of the transactivation status of AR, AR mutants or AR splice variants (Figure 6). Using the ENZ-resistant MR49F and androgen-independent LNCaP95 cells models, we show that ICRF187 and ICRF913 potently inhibit AR signaling (Figures 1–3), cancer cell proliferation (Figure 4) and CRPC xenograft growth (Figure 5). Catalytic Topo II inhibitors may therefore represent a novel class of non-LBD inhibitors of AR signaling beneficial for CRPC patients.

In contrast to AR pathway inhibitors, catalytic Topo II inhibitors target not only AR-mediated transcription initiation but also suppress cancer cell mitosis. Since a downstream effector of re-activated AR signalling in CRPC tumors is acceleration of tumor cell mitosis [6, 7], targeting both cancer cell cycling and AR transcriptional activity may more strongly inhibit CRPC growth. In support of this hypothesis, inhibitors to cyclin-dependent kinases are effective in delaying ENZ-resistance cancer cell growth [20]. Our results further indicated that co-treatment of Topo II inhibitors with ENZ results in stronger suppression to AR signaling (Figures 1–3) and CRPC cell growth and tumor growth (Figure 4–5).

The knowledge that Topo II is required for AR transcriptional activity may lead to two possible therapeutic strategies for prostate cancer patients. One possibility is to utilize super physiological doses of androgen in combination with Topo II poisons, as proposed previously [22], to capitalize on androgen-activated AR and AR-recruited Topo II to target promoters. However, due to the presence of Topo II poisons, AR-induced DNA double strand breaks cannot be fixed thereby leading to G2/M cell cycle arrest followed by apoptotic cell death. The potential risk would be that surviving cells could accumulate complex genomic rearrangement, adaptive DNA repair systems and greater heterogeneity. Additionally, AR transcriptional activity is enhanced by Topo II poisons (Figure S3). The other possible therapeutic strategy is applying AR pathway inhibitors with catalytic Topo II inhibitors to repress AR activation and AR-mediated transcription initiation. Since catalytic topo II inhibitors prevent DNA double strand breaks, they also cause cell cycle delay at the G2/M phases by interfering chromosome condensation and segregation during mitosis, and have lower chance to induce further genomic rearrangement.

In summary, we demonstrate that catalytic Topo II inhibitors can block AR signaling and inhibit tumor growth of castration-resistant xenografts, identifying catalytic Topo II inhibitors as potentially novel drugs to treat in patients with CRPC.

## MATERIALS AND METHODS

### Cell culture and reagents

Human LNCaP, 22RV1, VCaP, PC3 and 293T cell lines were from American Type Culture Collection. LNCaP C4-2, LNCaP95 and LNCaP(AI) were from Drs. Leland Chung (Cedars-Sinai), Alan Meeker (Johns Hopkins University) and Ralph Buttyan (Vancouver Prostate Centre). LNCaP95 and LNCaP(AI) cell were maintained in RPMI1640 medium with 5% charcoal stripped serum (CSS) (Hyclone) as we reported [23, 24]. MR49F cells were derived from LNCaP cells and cultured in medium containing 10uM of ENZ [25]. PC3(AR-V7) cells are PC3 cells induced exogenous AR-V7 protein by lentiviral approach. All cell lines had been either recently purchased or authenticated by short tandem repeat assays. AR protein levels in these prostate cancer cells were shown in Figure S1. R1881, ICRF187 and ICRF193, Merbarone, Aclacinomycin A and Genistein were purchased from Cedarlane (Burlington, Canada). ENZ was ordered from Haoyuan Chemexpress (Shanghai, China).

### Real-time PCR & immunoblotting

Real-time PCR assays were performed as described [26, 27]. Total RNA was extracted using Purelink RNA mini kit (Invitrogen). Real-time PCR was conducted in triplicates using Applied Biosystems7900HT with 5ng of cDNA, 1uM of each primer pair and SYBR green PCR master mix (Roche). All real-time PCR assays were carried out using three technical replicates and three independent cDNA syntheses. Western blotting assays were performed as we reported [28]. Experiments were repeated in three independent experiments and one representative blots were shown. Information on primers and antibodies were listed in Supplementary Table.

### Transfection and luciferase reporter assay

Plasmid DNA were transfected into cells using Lipofectmine 2000 (Invitrogen), while siRNA was transfected by siLentFect reagent (Bio-Rad). Luciferase activities were determined using the luciferin reagent (Promega) according to the manufacturer's protocol. Transfection efficiency was normalized to Renilla luciferase activities.

### Chromatin immunoprecipitation (ChIP) and fluorescence microscopy

ChIP assays were performed with AR antibody (Santa Cruz) as we described [23]. To determine AR subcellular localization, LNCaP cells stably expressing GFP-tagged AR were used. Cells were counterstained by DAPI and images were captured by using a Zeiss LSM780 confocal microscope (Carl Zeiss Instruments) as we reported [28]. AR subcellular localization was also confirmed by Western blotting nuclear and cytoplasmic extracts from cells transfected with Flag-tagged AR, AR mutants and the AR-V7 splice variant.

### MTS assays

Cell proliferation rates were measured by using the 3-(4, 5-dimethylthiazol-2-yl)-5-(3- carbo xymethoxyphenyl)-2-(4-sulfophenyl)-2Htetrazolium (Promega) assay according to the manufacture's protocol.

### Fluorescence-activated cell sorting (FACS)

Cell cycling was analyzed by FACS with 40ug/mL propidium iodide staining following the protocol (http://www.meduniwien.ac.at/user/johannes.schmid/PIstaining3.htm). Relative DNA contents from 10,000 cells were analyzed by FACSCanto II flow cytometer and BD FACSDiva software v5.0.3 (Becton Dickinson) as we reported [28].

### Human prostate cancer xenografts

To construct CRPC LNCaP xenografts, a total of 1×10^6^ LNCaP cells in 0.1mL Matrigel (Becton Dickinson Labware) were inoculated subcutaneously in bilateral flanks of 6-8 week old male athymic nude mice (Harlan Sprague Dawley Inc.). Tumor volume (V= L*W*D*0.5236) and body weight were measured weekly. Serum PSA levels were determined by ELISA. When serum PSA concentrations reached 50 ng/mL, mice were castrated. CRPC LNCaP tumors were defined when PSA levels relapsed to pre-castrated levels. Animals were randomly separated into four groups treated daily with control, 10mg/kg ENZ, 50mg/kg ICRF187 or 5mg/kg ENZ plus 25mg/kg ICRF187. To establish ENZ-resistant MR49F xenografts, castrated mice were inoculated subcutaneously with MR49F cells and treated with 10mg/kg ENZ daily till endpoint. When tumor volumes reached 100 mm^3^, mice were also treated daily with vehicle, 25mg/kg or 50mg/kg of ICRF187. Tumor volumes and PSA levels were measured. CRPC 22RV1 xenografts were constructed by inoculating 22RV1 cells subcutaneously in bilateral flanks of nude mice. When mice were castrated and tumors reached to 100 mm^3^, mice were treated daily with vehicle or 50mg/kg ICRF187. PC3 xenografts were constructed by inoculating PC3 cells subcutaneously in male athymic nude mice. When tumors reached 200 mm^3^, mice were treated daily with vehicle or 50mg/kg ICRF187. In all experiments, animals will be sacrificed if tumors mass is ~10% of the body weight or any endpoint situation according to the animal protocol. At the endpoint, tumors were harvested for evaluations of mRNA levels of AR targeted genes by real-time PCR. Tumors samples were also used to construct tissue microarray to measure Ki67 expressions by immunohistochemistry (IHC). All animal procedures were under the guidelines of the Canadian Council on Animal Care.

### Statistics

Statistical analysis was carried out using GraphPad Prism (version 6) with the level of significance set at *P*<0.05 as *, *P*<0.01 as ** and *P*<0.001 as ***. One-way ANOVA plus student's *t*-test were used to compare among groups when the means follows the normal distribution. Detail statistical analyses were addressed in each figure legend.

## SUPPLEMENTARY FIGURES AND TABLES



## References

[1] Visakorpi T, Hyytinen E, Koivisto P, Tanner M, Keinanen R, Palmberg C, Palotie A, Tammela T, Isola J, Kallioniemi OP (1995). In vivo amplification of the androgen receptor gene and progression of human prostate cancer. Nat Genet.

[2] Taplin ME, Bubley GJ, Shuster TD, Frantz ME, Spooner AE, Ogata GK, Keer HN, Balk SP (1995). Mutation of the androgen-receptor gene in metastatic androgen-independent prostate cancer. The New England journal of medicine.

[3] Locke JA, Guns ES, Lubik AA, Adomat HH, Hendy SC, Wood CA, Ettinger SL, Gleave ME, Nelson CC (2008). Androgen levels increase by intratumoral de novo steroidogenesis during progression of castration-resistant prostate cancer. Cancer research.

[4] Scher HI, Fizazi K, Saad F, Taplin ME, Sternberg CN, Miller K, de Wit R, Mulders P, Chi KN, Shore ND, Armstrong AJ, Flaig TW, Flechon A, Mainwaring P, Fleming M, Hainsworth JD (2012). Increased survival with enzalutamide in prostate cancer after chemotherapy. The New England journal of medicine.

[5] de Bono JS, Logothetis CJ, Molina A, Fizazi K, North S, Chu L, Chi KN, Jones RJ, Goodman OB, Saad F, Staffurth JN, Mainwaring P, Harland S, Flaig TW, Hutson TE, Cheng T (2011). Abiraterone and increased survival in metastatic prostate cancer. The New England journal of medicine.

[6] Wang Q, Li W, Zhang Y, Yuan X, Xu K, Yu J, Chen Z, Beroukhim R, Wang H, Lupien M, Wu T, Regan MM, Meyer CA, Carroll JS, Manrai AK, Janne OA (2009). Androgen receptor regulates a distinct transcription program in androgen-independent prostate cancer. Cell.

[7] Cai C, He HH, Chen S, Coleman I, Wang H, Fang Z, Nelson PS, Liu XS, Brown M, Balk SP (2011). Androgen receptor gene expression in prostate cancer is directly suppressed by the androgen receptor through recruitment of lysine-specific demethylase 1. Cancer cell.

[8] Haffner MC, Aryee MJ, Toubaji A, Esopi DM, Albadine R, Gurel B, Isaacs WB, Bova GS, Liu W, Xu J, Meeker AK, Netto G, De Marzo AM, Nelson WG, Yegnasubramanian S (2010). Androgen-induced TOP2B-mediated double-strand breaks and prostate cancer gene rearrangements. Nat Genet.

[9] Ju BG, Lunyak VV, Perissi V, Garcia-Bassets I, Rose DW, Glass CK, Rosenfeld MG (2006). A topoisomerase IIbeta-mediated dsDNA break required for regulated transcription. Science.

[10] Lin C, Yang L, Tanasa B, Hutt K, Ju BG, Ohgi K, Zhang J, Rose DW, Fu XD, Glass CK, Rosenfeld MG (2009). Nuclear receptor-induced chromosomal proximity and DNA breaks underlie specific translocations in cancer. Cell.

[11] Hughes C, Murphy A, Martin C, Fox E, Ring M, Sheils O, Loftus B, O'Leary J (2006). Topoisomerase II-alpha expression increases with increasing Gleason score and with hormone insensitivity in prostate carcinoma. Journal of clinical pathology.

[12] Schmidt F, Knobbe CB, Frank B, Wolburg H, Weller M (2008). The topoisomerase II inhibitor, genistein, induces G2/M arrest and apoptosis in human malignant glioma cell lines. Oncology reports.

[13] Bower JJ, Zhou Y, Zhou T, Simpson DA, Arlander SJ, Paules RS, Cordeiro-Stone M, Kaufmann WK (2010). Revised genetic requirements for the decatenation G2 checkpoint: the role of ATM. Cell cycle.

[14] Munoz P, Baus F, Piette J (2001). Ku antigen is required to relieve G2 arrest caused by inhibition of DNA topoisomerase II activity by the bisdioxopiperazine ICRF-193. Oncogene.

[15] Morris SK, Baird CL, Lindsley JE (2000). Steady-state and rapid kinetic analysis of topoisomerase II trapped as the closed-clamp intermediate by ICRF-193. J Biol Chem.

[16] Ishida R, Sato M, Narita T, Utsumi KR, Nishimoto T, Morita T, Nagata H, Andoh T (1994). Inhibition of DNA topoisomerase II by ICRF-193 induces polyploidization by uncoupling chromosome dynamics from other cell cycle events. The Journal of cell biology.

[17] Taylor BS, Schultz N, Hieronymus H, Gopalan A, Xiao Y, Carver BS, Arora VK, Kaushik P, Cerami E, Reva B, Antipin Y, Mitsiades N, Landers T, Dolgalev I, Major JE, Wilson M (2010). Integrative genomic profiling of human prostate cancer. Cancer cell.

[18] Shapiro AB, Austin CA (2014). A high-throughput fluorescence anisotropy-based assay for human topoisomerase II beta-catalyzed ATP-dependent supercoiled DNA relaxation. Analytical biochemistry.

[19] Joseph JD, Lu N, Qian J, Sensintaffar J, Shao G, Brigham D, Moon M, Maneval EC, Chen I, Darimont B, Hager JH (2013). A clinically relevant androgen receptor mutation confers resistance to second-generation antiandrogens enzalutamide and ARN-509. Cancer discovery.

[20] Korpal M, Korn JM, Gao X, Rakiec DP, Ruddy DA, Doshi S, Yuan J, Kovats SG, Kim S, Cooke VG, Monahan JE, Stegmeier F, Roberts TM, Sellers WR, Zhou W, Zhu P (2013). An F876L mutation in androgen receptor confers genetic and phenotypic resistance to MDV3100 (enzalutamide). Cancer discovery.

[21] Antonarakis ES, Lu C, Wang H, Luber B, Nakazawa M, Roeser JC, Chen Y, Mohammad TA, Fedor HL, Lotan TL, Zheng Q, De Marzo AM, Isaacs JT, Isaacs WB, Nadal R, Paller CJ (2014). AR-V7 and resistance to enzalutamide and abiraterone in prostate cancer. The New England journal of medicine.

[22] Denmeade SR, Isaacs JT (2010). Bipolar androgen therapy: the rationale for rapid cycling of supraphysiologic androgen/ablation in men with castration resistant prostate cancer. The Prostate.

[23] Xie N, Cheng H, Lin D, Liu L, Yang O, Jia L, Fazli L, Gleave ME, Wang Y, Rennie P, Dong X (2014). The expression of glucocorticoid receptor is negatively regulated by active androgen receptor signaling in prostate tumors. Int J Cancer.

[24] Liu LL, Xie N, Sun S, Plymate S, Mostaghel E, Dong X (2014). Mechanisms of the androgen receptor splicing in prostate cancer cells. Oncogene.

[25] Toren PJ, Kim S, Pham S, Mangalji A, Adomat H, Tomlinson Guns ES, Zoubeidi A, Moore W, Gleave ME (2014). Anti-Cancer Activity of a Novel Selective Cyp17a1 Inhibitor in Pre-Clinical Models of Castrate Resistant Prostate Cancer. Molecular cancer therapeutics.

[26] Yu Y, Lee JS, Xie N, Li E, Hurtado-Coll A, Fazli L, Cox M, Plymate S, Gleave M, Dong X (2014). Prostate stromal cells express the progesterone receptor to control cancer cell mobility. PloS one.

[27] Liu L, Li Y, Xie N, Shynlova O, Challis JR, Slater D, Lye S, Dong X (2013). Proliferative action of the androgen receptor in human uterine myometrial cells--a key regulator for myometrium phenotype programming. The Journal of clinical endocrinology and metabolism.

[28] Yu Y, Liu L, Xie N, Xue H, Fazli L, Buttyan R, Wang Y, Gleave M, Dong X (2013). Expression and function of the progesterone receptor in human prostate stroma provide novel insights to cell proliferation control. The Journal of clinical endocrinology and metabolism.

