# Correction: Catalytic inhibitors of DNA topoisomerase II suppress the androgen receptor signaling and prostate cancer progression

**DOI:** 10.18632/oncotarget.28692

**Published:** 2025-02-12

**Authors:** Haolong Li, Ning Xie, Martin E. Gleave, Xuesen Dong

**Affiliations:** ^1^The Vancouver Prostate Centre, Department of Urologic Sciences, University of British Columbia, Canada

**This article has been corrected:** Oncotarget has investigated concerns regarding duplicate images in this paper. In Figure 3, the tubulin band in panel 3D is a duplicate of the H3 band in panel 3C. Additionally, the Actin band is a duplicate of one shown in Figure 4C of an earlier article that includes two authors in common with the Oncotarget paper [1]. We also found duplication in Supplementary Figure 1 (AR-V7 western blot of three LANCaP cell lines) which overlaps with WB band in Figure 7C of [1]. The corresponding author of both these articles, Dr. Xuesen Dong, has stated: “The reason for these mistakes was that Dr. Haolong Li had been working on two publications (Oncotarget and Cell Death and Disease) at the same time. Each project involved a large amount of western blotting assays; all images for the loading controls look very similar and were easily misplaced. Regardless, these minor mistakes did not affect the conclusion we have drawn.” The authors provided original western blots with date stamps for the corrected figures and stated that Figure 3A Actin (2 h treatment), Figure 3D Tubulin (second panel, 293T cells transfected with plasmid encoding AR(F876L) and Supplementary Figure 1 AR-V7 blot were misplaced during the figure assembly. The corrected Figure 3 and Supplementary Figure 1, obtained using the original data, are shown below. The authors declare that these corrections do not change the results or conclusions of this paper.

Original article: Oncotarget. 2015; 6:20474–20484. 20474-20484. https://doi.org/10.18632/oncotarget.4105

**Figure 3:**
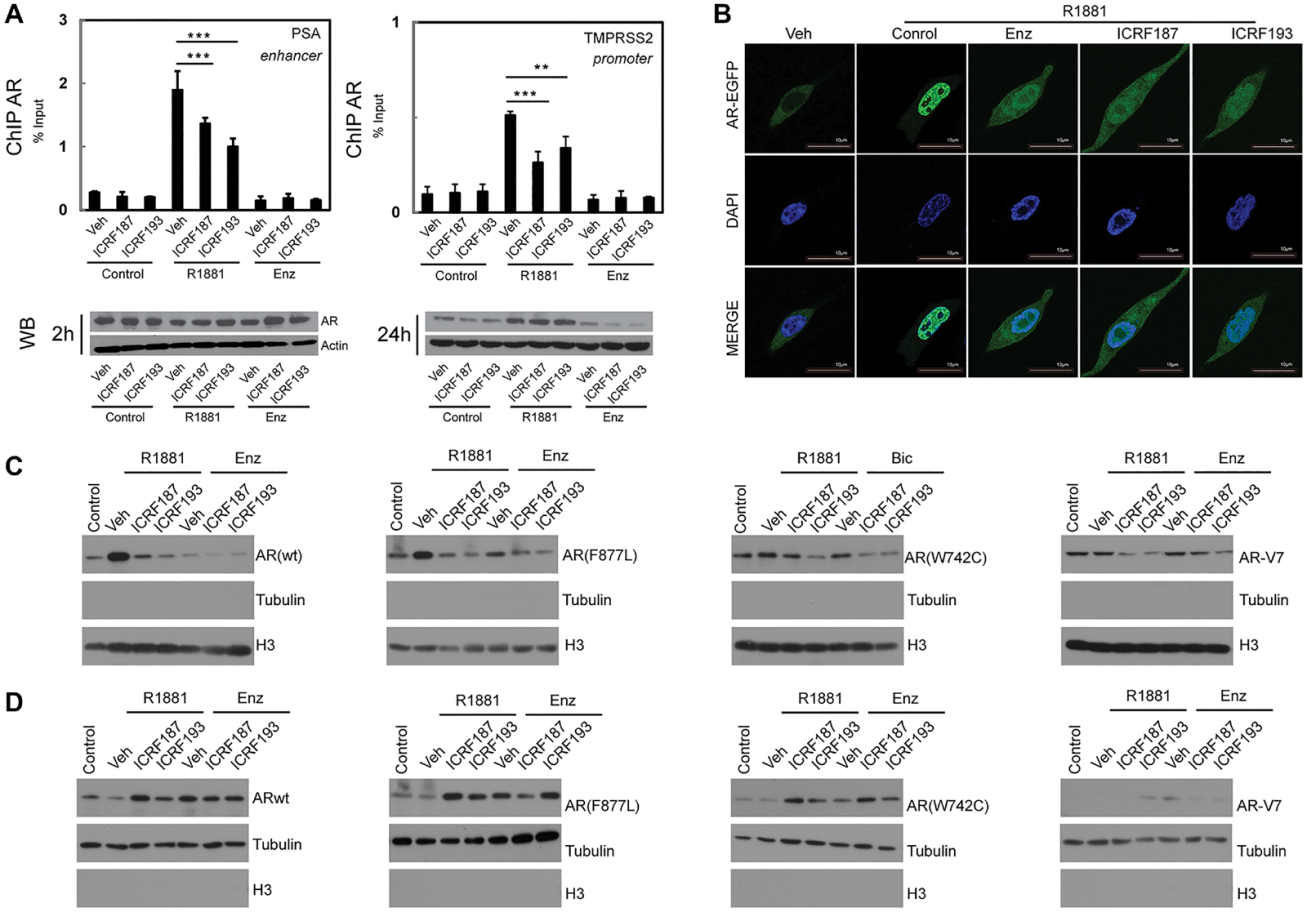
ICRF187 and ICRF193 inhibit AR recruitment to target promoters and AR nuclear localization. (**A**) LNCaP cells were cultured in RPMI1640 medium containing 5% CSS and treated with vehicle, 1 uM of ICRF187 or 1 uM of ICRF193 in addition to vehicle, 10 nM of R1881 or 10 uM of ENZ treatment for 2 hours. Three independent ChIP experiments were performed using the AR antibody. Precipitated DNA fragment were used as templates to amplify the PSA enhancer and the TMPRSS2 promoter by real-time PCR. Data represented mean ± SEM (*n* = 3) and plotted as percentage of input. *P* < 0.01 ** and *P* < 0.001 as *** (student’s *t*-test). AR protein levels under 2 and 24 hour treatment were detected by Western blotting. (**B**) LNCaP cells expressing EGFP-AR were cultured in RPMI1640 medium containing 5% CSS. Cells were treated with vehicle, 10 nM of R1881, 10 nM of R1881 plus 10 μM of ENZ, 10 nM of R1881 plus 1uM of ICRF187, or 10 nM of R1881 plus 1 uM of ICRF193 for 6 hours. Cells were then fixed with 4% paraformaldehyde and mounted with DAPI. Representative confocal microscopic images showed AR localization (Green) and nucleus (Blue). (**C**, **D**) 293T cells were transfected with plasmids encoding wild type AR, AR(F876L), AR(W741C) and AR-V7. Cells were treated with vehicle, 1 uM of ICRF187 or 1 uM of ICRF193 in addition to 10 nM of R1881, 10uM of ENZ or 10 uM of bicalutamide for 24 hours. Nuclear (C) and cytosol (D) protein extract were immunoblotted with AR, tubulin and Histone H3 antibodies. Three independent experiments were performed and one set of Western blotting images are presented.

**Supplementary Figure 1:**
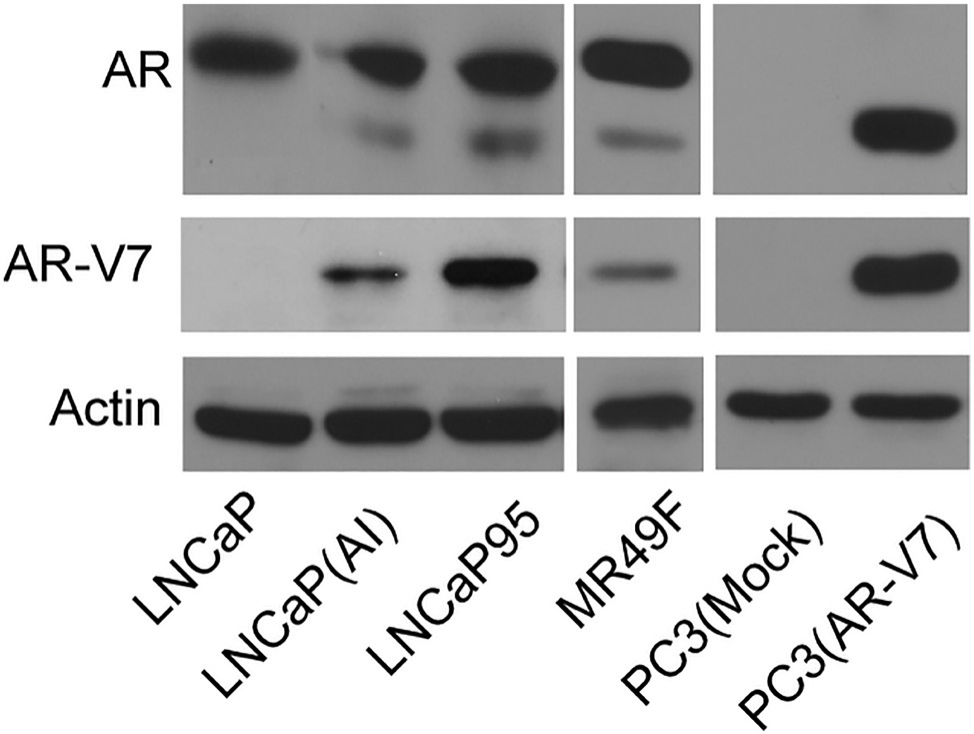
AR and AR-V7 protein levels in LNCaP, LNCaP(AI), LNCaP95, MR49F, PC3(mock) and PC3(AR-V7) cell lines were detected by Western blotting with AR (N-20) and AR-V7 antibodies. Beta actin was used as loading control.
