# Supplementary material for: Catalytic inhibitors of DNA topoisomerase II suppress the androgen receptor signaling and prostate cancer progression
Source: Oncotarget. 2015 May 12;6(24):20474–84. doi: 10.18632/oncotarget.4105 (PMC4653019; doi:10.18632/oncotarget.4105)
Supplement: Supplementary file 1 [file oncotarget-06-20474-s001.pdf]

Catalytic inhibitors of DNA topoisomerase II suppress the androgen receptor signaling and prostate cancer progression

Supplementary Material

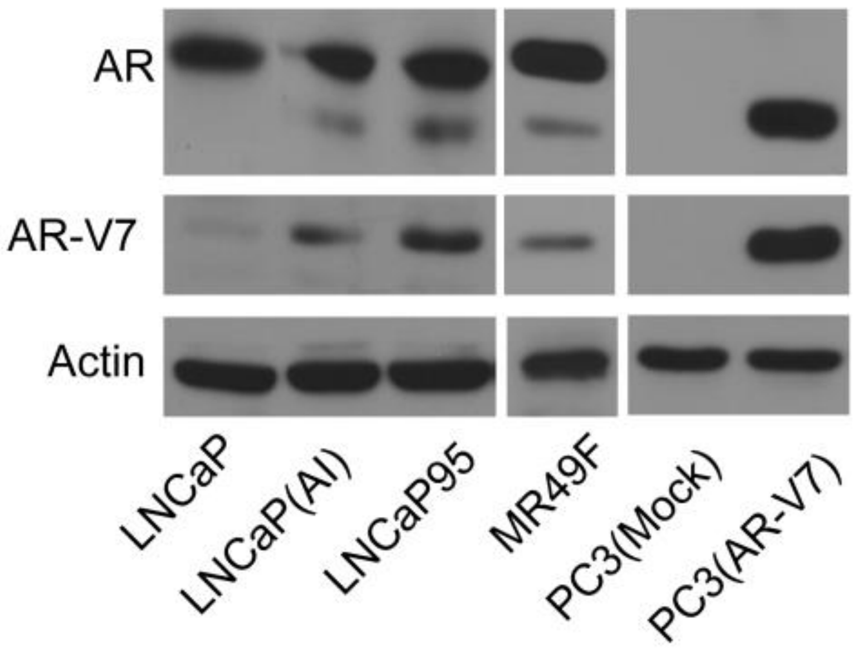

Figure S1: AR and AR-V7 protein levels in LNCaP, LNCaP(AI), LNCaP95, MR49F, PC3(mock) and PC3(AR-V7) cell lines were detected by Western blotting with AR (N-20) and AR-V7 antibodies. Beta actin was used as loading control.

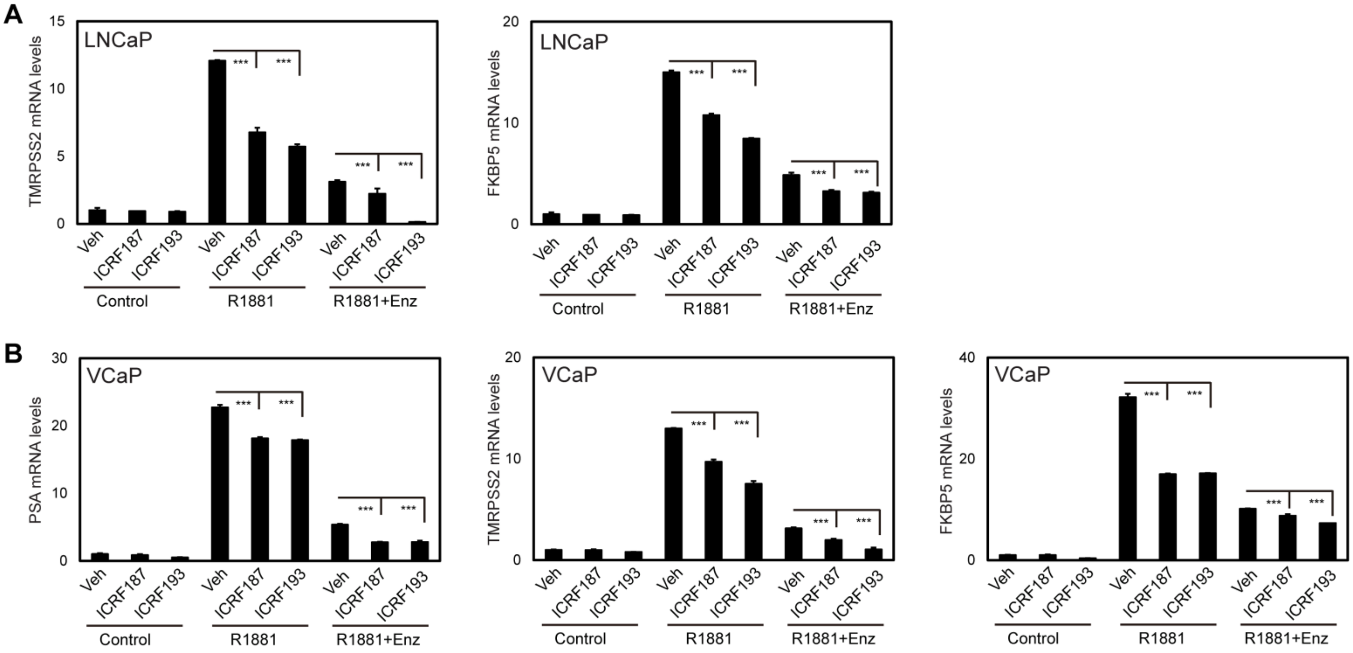

Figure S2: (A) LNCaP and **(B)** VCaP cells were cultured in medium containing 5% CSS. Cells were treated with vehicle, 1nM of R1881 or 1nM of R1881 plus 5uM of ENZ for 24 hours. Cells were also co-treated with vehicle, 1uM of ICRF187 or 1uM of ICRF193 as indicated. Relative RNA levels of PSA, TMPRSS2 and FKBP5 to GAPDH were measured by real-time PCR from three independent experiments. Data represent mean ± SEM (n=3) with P<0.01 as ** and P<0.001 as *** (student's t-test).

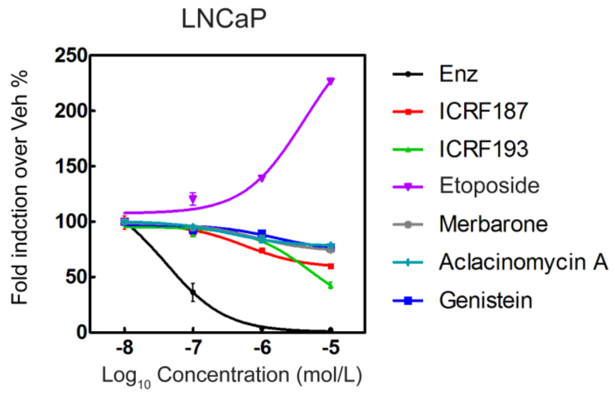

Figure S3: LNCaP cells were transfected with a PSA-luciferase reporter and treated with 1nM of R1881. Cells were also treated with vehicle or 0.01-10uM of ENZ, ICRF187, ICRF193, Etoposide, Merbarone, Aclacinomycin A or Genistein for 24 hours. Relative luciferase activities were calibrated with Renilla from three independent experiments and were presented as mean ± SEM (n=3). Values from vehicle treatment were set as 100%.

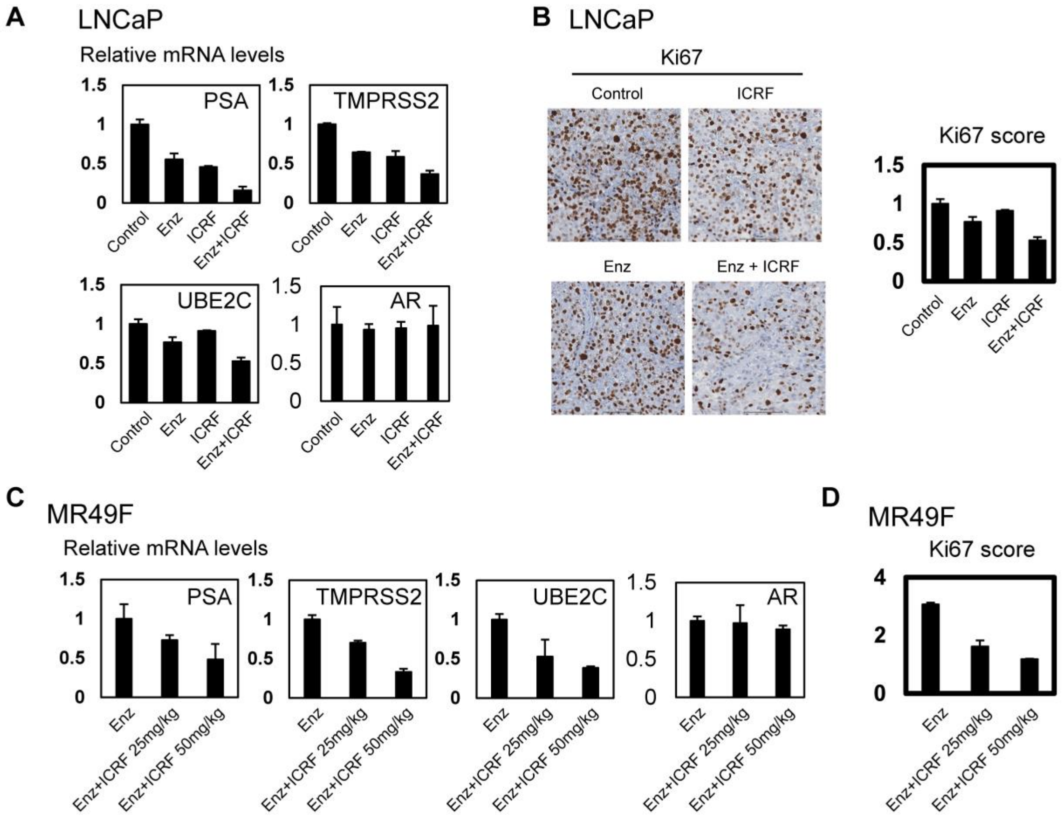

Figure S4: (A) PSA, TMPRSS2, UBE2C and AR mRNA levels from CRPC LNCaP xenografts treated with ENZ and or ICRF187 were measured by real-time PCR. **(B)** Ki67 histology score was determined. **(C)** PSA, TMPRSS2, UBE2C and AR mRNA levels from ENZ-resistant MR49F xenografts treated with ENZ and or ICRF187 were measured by real-time PCR. **(D)** Ki67 histology score was determined.

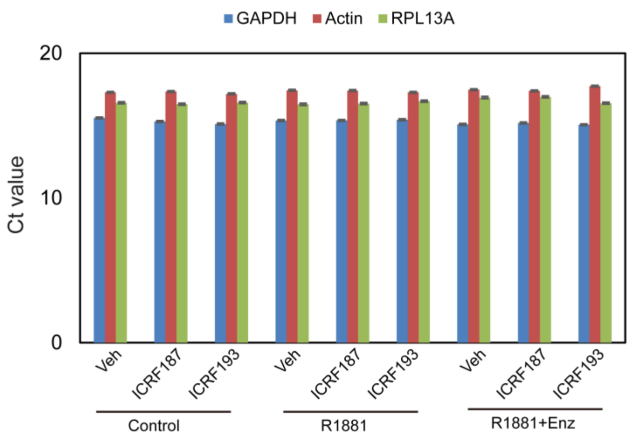

Figure S5: LNCaP cells were cultured in medium containing 5% CSS. Cells were treated with vehicle, 1nM of R1881 or 1nM of R1881 plus 5uM of ENZ for 24 hours. Cells were also co-treated with vehicle, 1uM of ICRF187 or 1uM of ICRF193 as indicated. Ct values of GAPDH, beta actin and RPL13A were measured by real-time PCR.

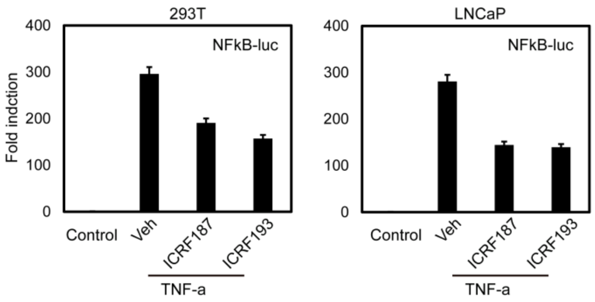

Figure S6: (A) 293T and **(B)** LNCaP cells were transiently transfected with an NFkB-luciferase reporter (Invitrogen). Cells were then treated with either vehicle, 100ng/ml TNFa, 100ng/ml TNFa plus 10uM of ICRF187 or 100ng/ml TNF-a plus 2uM of ICRF193 for 6 hours. Luciferase activities were calibrated with Renilla from three independent experiments and presented as mean ± SEM (n=3). Values from vehicle treatment were set as 1.

A Cell population ditrubution without synchronization

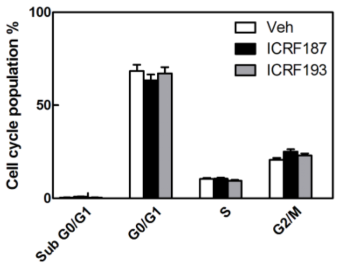

B

LNCaP	G0/G1	G2/M
Serum Starve	87.6±3.8%	4.5±2.3%
Nocodazole	21.0±3.8%	61.2±4.3%

LNCaP95	G0/G1	G2/M
Serum Starve	80.2±5.8%	10.8±3.3%
Nocodazole	26.7.0±4.8%	50.4±6.3%

Figure S7: (A) none synchronised LNCaP cells were treated with vehicle, 10uM of ICRF187 or 2uM of ICRF193 and used to perform FACS assays to determine cell populations at G0/G1, S and G2/M phases. **(B)** Cell cycling were **s**ynchronized by either serum starve or nocodazole as described in material and method section. Cell populations at G0/G1 and G2/M phases were measured by FACS assays.

A

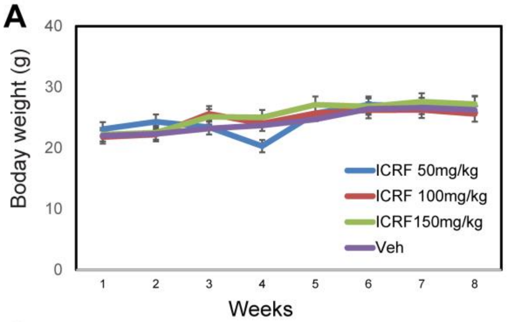

B

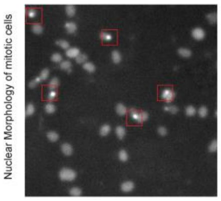

	Vehicle	ICRF187	ICRF193
LNCaP	20.6±5.8/100	32.6±7.8/100	37.5±3.8/100
LNCaP95	30.5±4.4/100	37.4±3.8/100	32.5±2.8/100

Mitotic Cell number/Total cell number

C

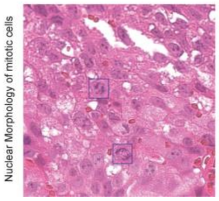

	Vehicle	Enz	ICRF	Enz+ICRF
LNCaP	4.9±0.8/1000	2.6±1.7/1000	5.7±2.8/1000	7.5±4.7/1000

	Enz	Enz+ICRF 25mg/kg	Enz+ICRF 50mg/kg
MR49F	8.6±5.8/1000	9.1±4.8/1000	9.5±5.8/1000

Mitotic Cell number/Total cell number

Figure S8: (A) Male nude mice were treated with vehicle, 50mg/kg ICRF187, 100mg/kg ICRF187 or 150mg/kg ICRF187 (n=5/group) for 2 months. Body weight was measured weekly. Data was shwon as mean ± SEM (n=5). **(B)** LNCaP and LNCaP95 cell cycling were synchronized by nocodazole and released for 1.5 hours for LNCaP cells and 2 hours for LNCaP95 cells as described in figure 4C. Cells were fixed by 4% PFA and stained with DAPI. None overlapping fields (n=10) under 400X magnification were selected to count mitotic cell numbers. **(C)** LNCaP and MR49F xenografts from Figure 5 were stained with H&E. Five none overlapping fields from each tumor slide under 400X magnification were selected. Total cell numbers and mitotic cell numbers were counted manually with a mechanical tabulator.

Supplementary table

Real-time qPCR primers:

Topo IIb F	5'-AGC CAT TGA CGC AGT TCA TGT-3'
Topo IIb R	5'-CCT GGC ACA AAG GTA ACC TCC-3'
GAPDH F	5'-GGACCTGACCTGCCGTCTAGAA-3'
GAPDH R	5'-GGTGTCGCTGTTGAAGTCAGAG-3'
PSA F	5'-AGTGCGAGAAGCATTCCCAAC-3'
PSA R	5'-CCAGCAAGATCACGCTTTTGT T-3'
TMPSS2F	5'-CAGGAGTGTACGGGAATGTGATGGT-3'
TMPSS2 R	5'-GATTAGCCGTCTGCCCTCATTTGT-3'
UBE2C F	5' AGT GGC TAC CCT TAC AAT GCG 3'
UBE2C R	5' TTA CCC TGG GTG TCC ACG TT 3'
ACTIN F	5'-GGA CTT CGA GCA AGA GAT GG -3'
ACTIN R	5'-AGC ACT GTG TTG GCG TAC AG -3'
FKBP5 F	5'-AATGGTGAGGAAACGCCGATG -3'
FKBP5 R	5'-TCGAGGGAATTTTAGGGAGACT -3'
AR F	5'-CCAGGGACCATGTTTTGCC -3'
AR R	5'-CGAAGACGACAAGATGGACAA -3'
RPL13A F	5'-GCCATCGTGGCTAAACAGGTA -3'
RPL13A R	5'-GTTGGTGTTCATCCGCTTGC -3'

siRNA:

siRNA	Supplier and Cat No.
Control	Santa Cruz sc-37007
Topo IIb	Santa Cruz sc-36697

Primers for site-directed mutagenesis for AR

AR F876L F	5'-CGA GAG AGC TGC ATC AGT TAA CTT TTG-3'
AR F876L R	5'-AGC AGG TCA AAA GTT AAC TGA TGC AG-3'
AR F876L/T877A F	5'-CAG CTC GCT TTT GAC CTG CTA ATC AAG TCA CAC ATG-3'
AR F876L/T877A R	5'-ATG CAG CTC TCT CGC AAT AGG CTG CAC GGA G-3'
AR W741C F	5'-TCC TGC ATG GGG CTC ATG GTG TTT GCC ATG GGC TGG-3'
AR W741C R	5'-GTA CTG AAT GAC AGC CAT CTG GTC GTC CAC-3'

CHIP Primers:

PSA enhancer F	5'-TGG GAC AAC TTG CAA ACC TG-3'
PSA enhancer R	5'-CCA GAG TAG GTC TGT TTT CAA TCC A-3'
TMPRSS2 F	5'-TGG TCC TGG ATG ATA AAA AAA GTT T-3'
TMPRSS2 R	5'-GAC ATA CGC CCC ACA ACA GA-3'

Antibody Information

Antibody	Clone ID	Cat No.	Supplier
AR	N-20	sc-816	Santa Cruz Biotech
AR-V7		AG10008	Precision Antibody
Actin		A2066	Sigma
Tubulin	11H10	#2125	Cell Signaling
Histone H3		Ab1791	Abcam
Topo IIβ	H-286	sc-13059	Santa Cruz Biotech
